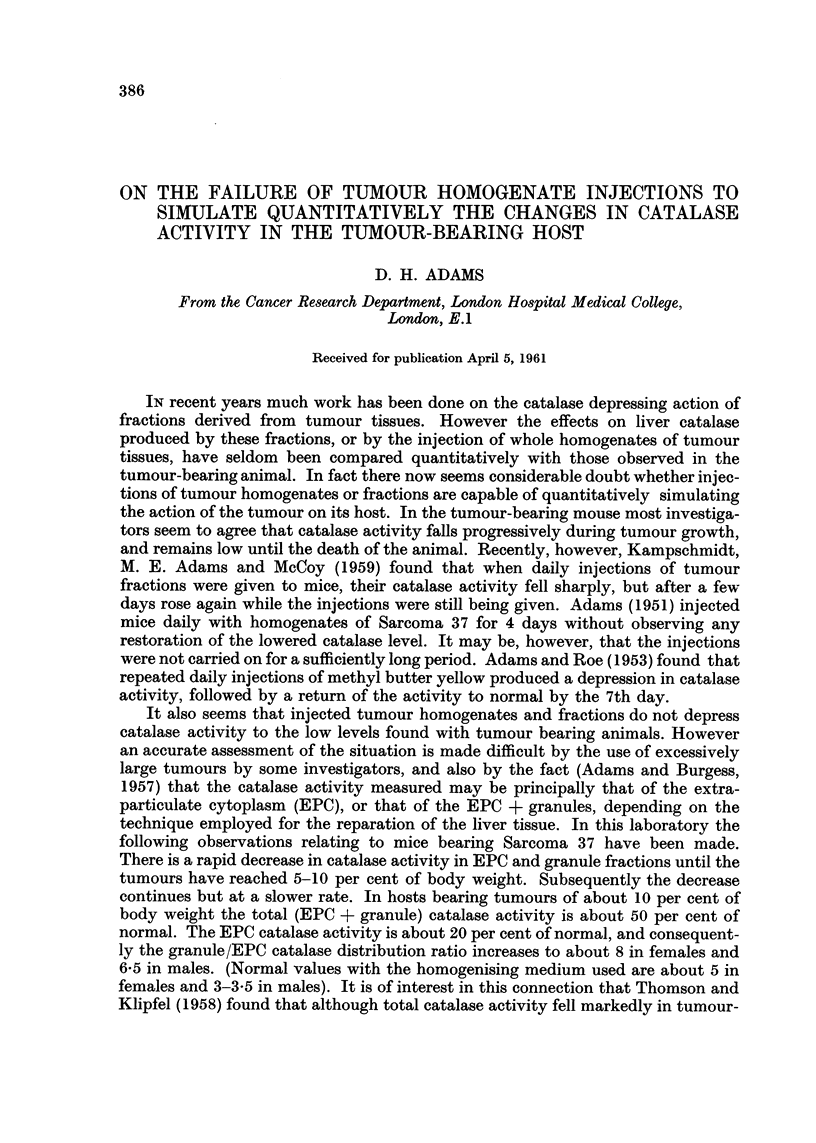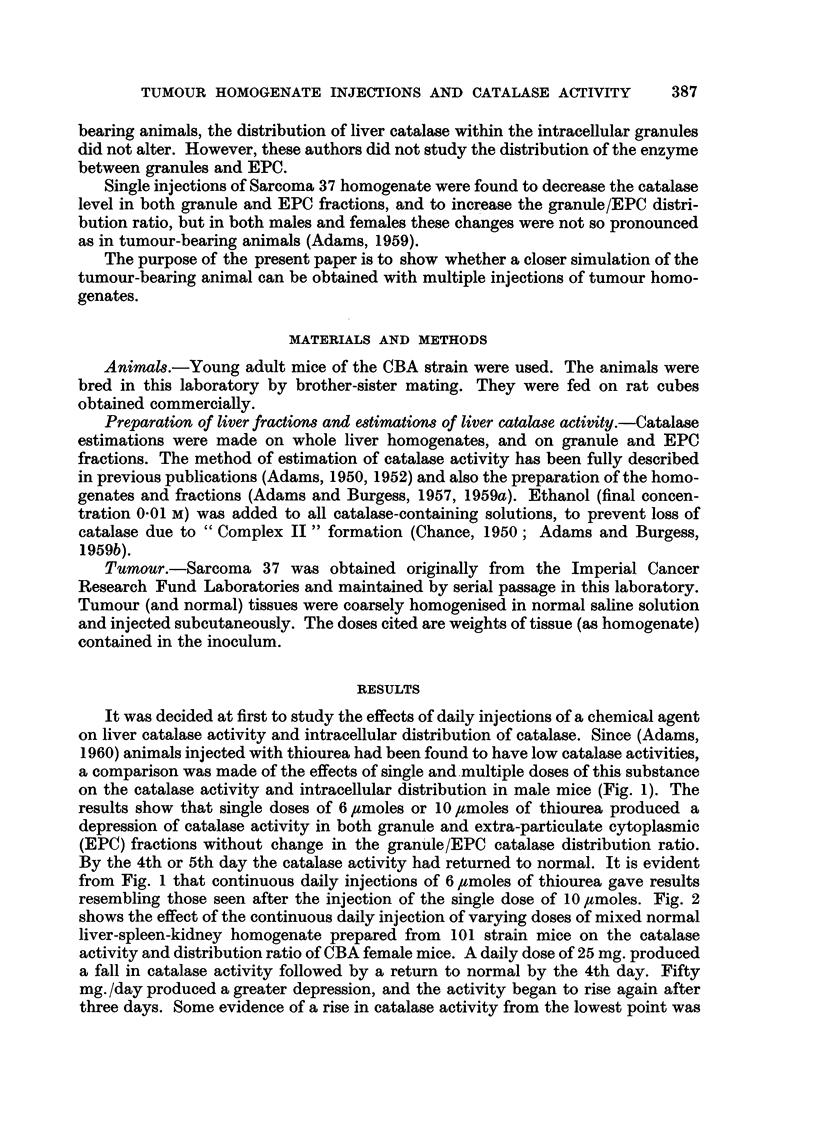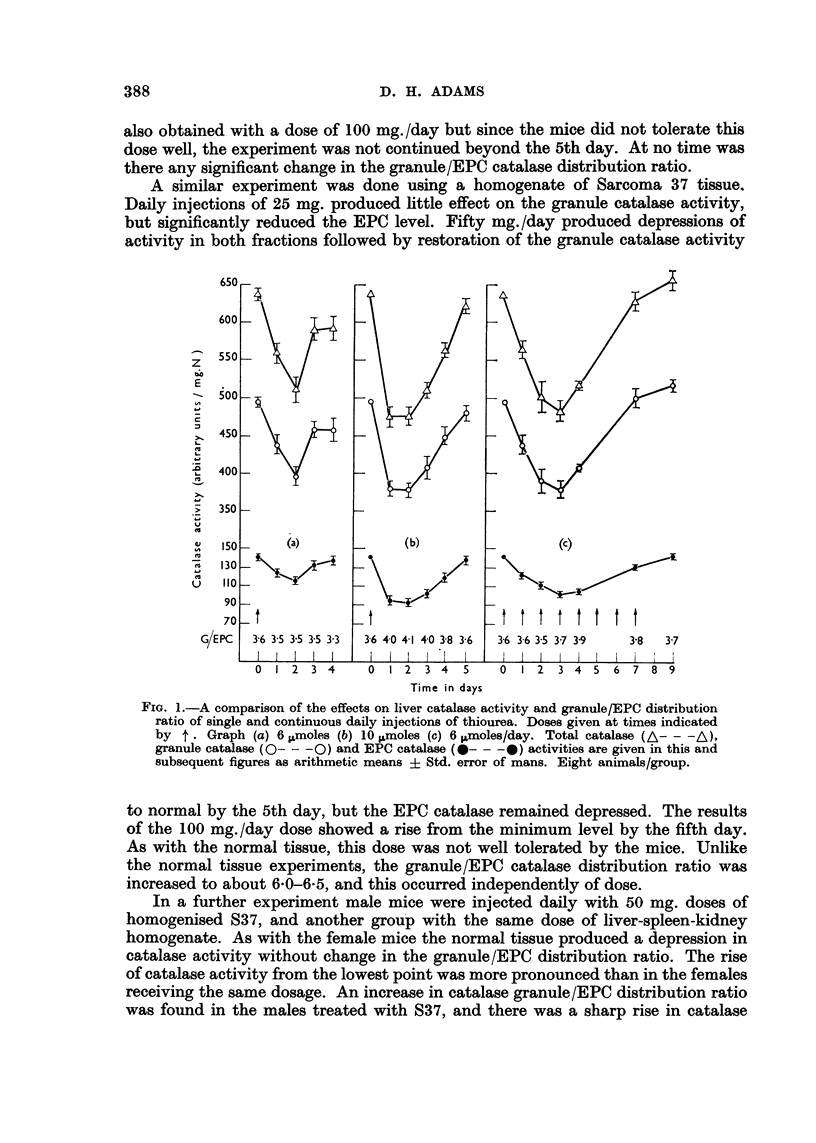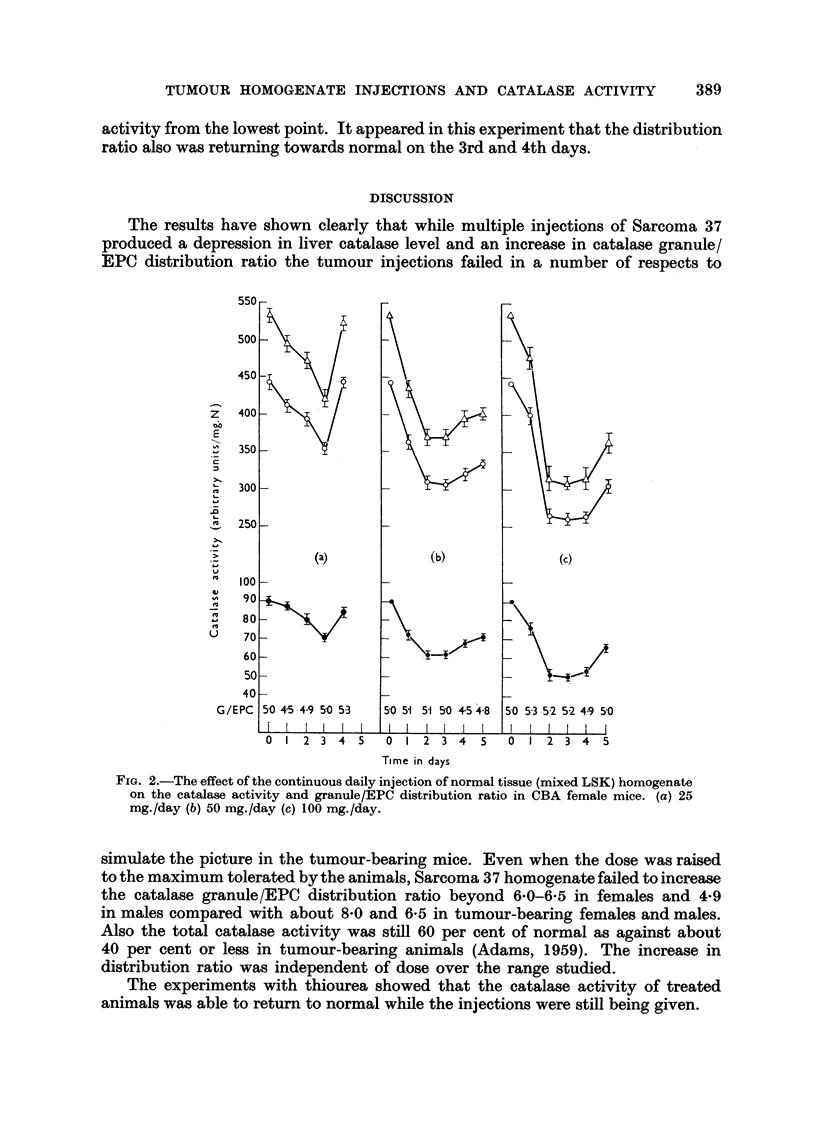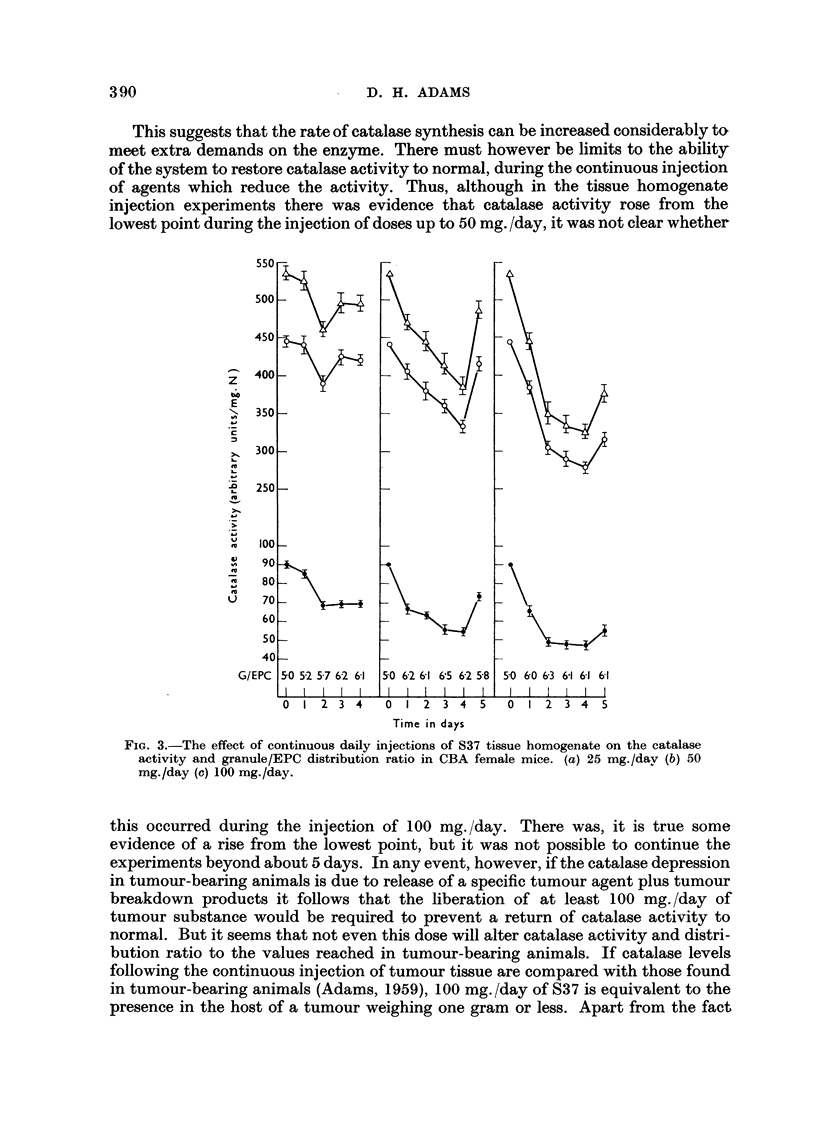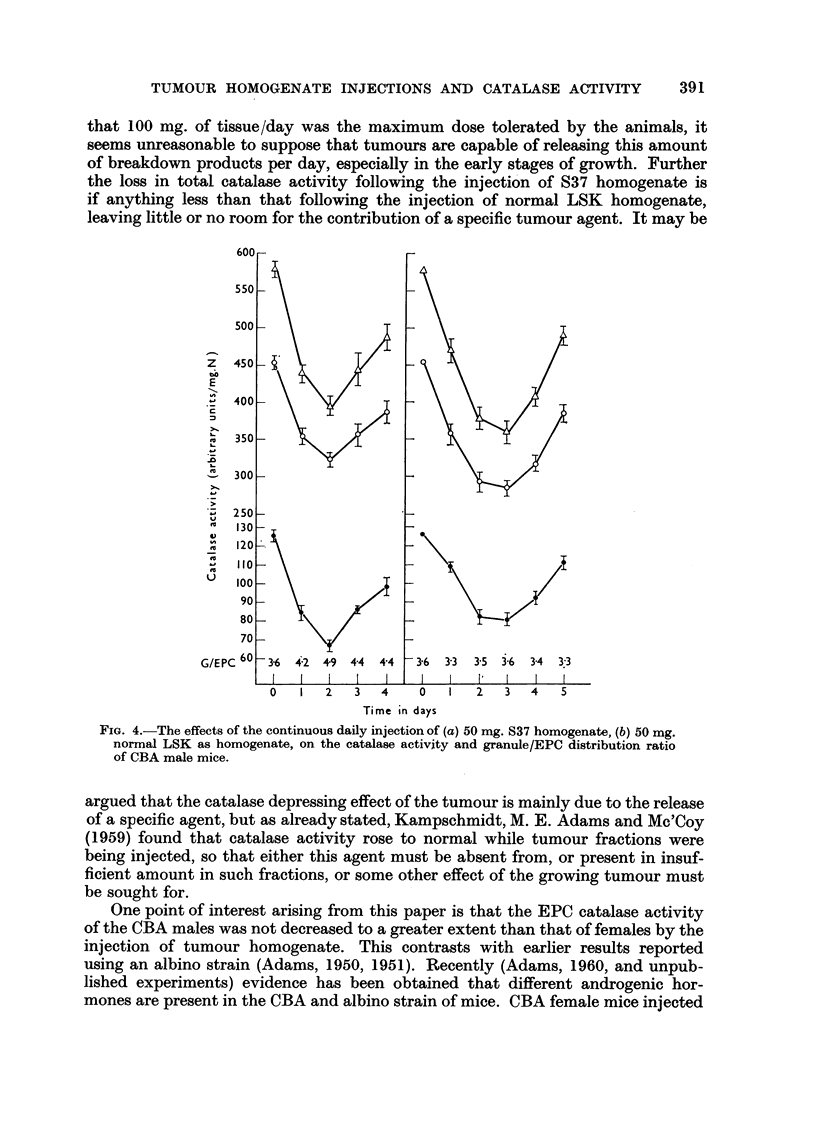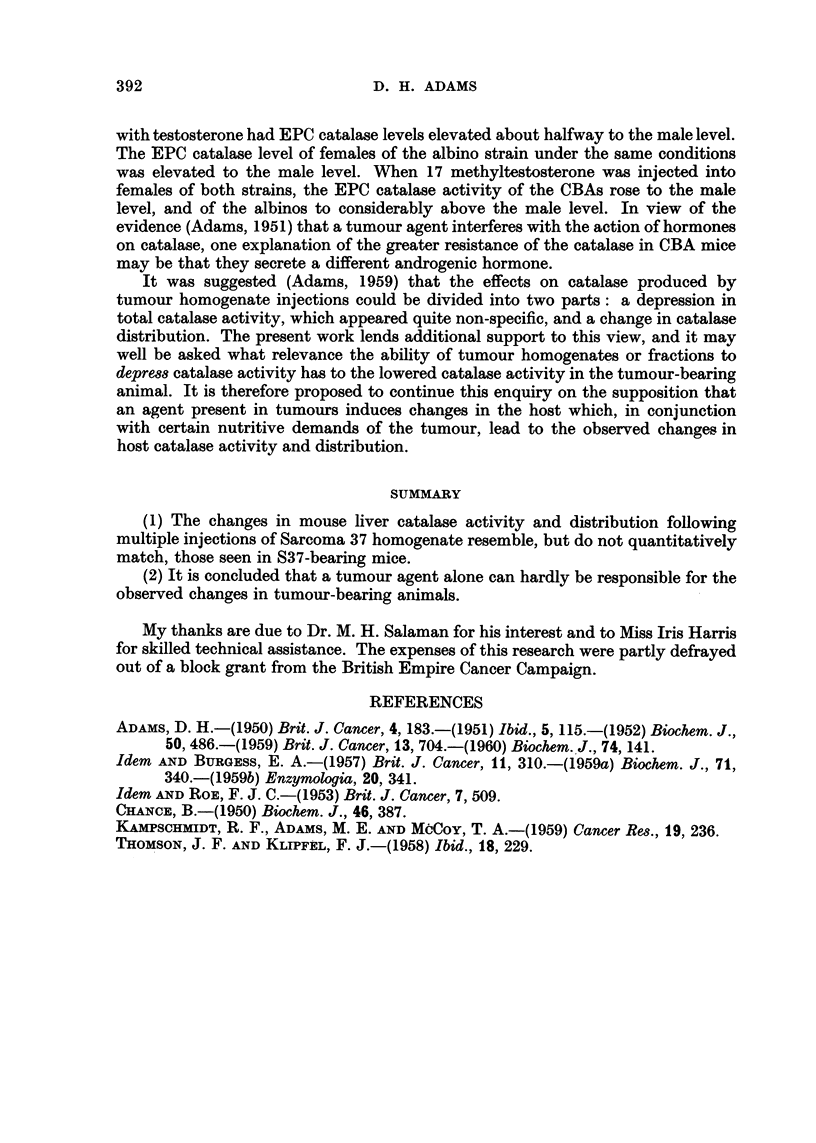# On the Failure of Tumour Homogenate Injections to Simulate Quantitatively the Changes in Catalase Activity in the Tumour-bearing host

**DOI:** 10.1038/bjc.1961.49

**Published:** 1961-06

**Authors:** D. H. Adams


### ON THE FAILURE OF TUMOUR HOMOGENATE INJECTIONS TO SIMULATE QUANTITATIVELY THE CHANGES IN CATALASE ACTIVITY IN THE TUMOUR-BEARING HOST

D. H. ADAMS

*From the Cancer Research Department, London Hospital Medical College, London, E.1*

Received for publication April 5, 1961

IN recent years much work has been done on the catalase depressing action of fractions derived from tumour tissues. However the effects on liver catalase produced by these fractions, or by the injection of whole homogenates of tumour tissues, have seldom been compared quantitatively with those observed in the tumour-bearing animal. In fact there now seems considerable doubt whether injections of tumour homogenates or fractions are capable of quantitatively simulating the action of the tumour on its host. In the tumour-bearing mouse most investigators seem to agree that catalase activity falls progressively during tumour growth, and remains low until the death of the animal. Recently, however, Kampschmidt, M. E. Adams and McCoy (1959) found that when daily injections of tumour fractions were given to mice, their catalase activity fell sharply, but after a few days rose again while the injections were still being given. Adams (1951) injected mice daily with homogenates of Sarcoma 37 for 4 days without observing any restoration of the lowered catalase level. It may be, however, that the injections were not carried on for a sufficiently long period. Adams and Roe (1953) found that repeated daily injections of methyl butter yellow produced a depression in catalase activity, followed by a return of the activity to normal by the 7th day.

It also seems that injected tumour homogenates and fractions do not depress catalase activity to the low levels found with tumour bearing animals. However an accurate assessment of the situation is made difficult by the use of excessively large tumours by some investigators, and also by the fact (Adams and Burgess, 1957) that the catalase activity measured may be principally that of the extra-particulate cytoplasm (EPC), or that of the EPC + granules, depending on the technique employed for the reparation of the liver tissue. In this laboratory the following observations relating to mice bearing Sarcoma 37 have been made. There is a rapid decrease in catalase activity in EPC and granule fractions until the tumours have reached 5–10 per cent of body weight. Subsequently the decrease continues but at a slower rate. In hosts bearing tumours of about 10 per cent of body weight the total (EPC + granule) catalase activity is about 50 per cent of normal. The EPC catalase activity is about 20 per cent of normal, and consequently the granule/EPC catalase distribution ratio increases to about 8 in females and 6·5 in males. (Normal values with the homogenising medium used are about 5 in females and 3–3·5 in males). It is of interest in this connection that Thomson and Klipfel (1958) found that although total catalase activity fell markedly in tumourbearing animals, the distribution of liver catalase within the intracellular granules did not alter. However, these authors did not study the distribution of the enzyme between granules and EPC.

Single injections of Sarcoma 37 homogenate were found to decrease the catalase level in both granule and EPC fractions, and to increase the granule/EPC distribution ratio, but in both males and females these changes were not so pronounced as in tumour-bearing animals (Adams, 1959).

The purpose of the present paper is to show whether a closer simulation of the tumour-bearing animal can be obtained with multiple injections of tumour homogenates.

### MATERIALS AND METHODS

*Animals.*—Young adult mice of the CBA strain were used. The animals were bred in this laboratory by brother-sister mating. They were fed on rat cubes obtained commercially.

*Preparation of liver fractions and estimations of liver catalase activity.*—Catalase estimations were made on whole liver homogenates, and on granule and EPC fractions. The method of estimation of catalase activity has been fully described in previous publications (Adams, 1950, 1952) and also the preparation of the homogenates and fractions (Adams and Burgess, 1957, 1959*a*). Ethanol (final concentration 0·01 M) was added to all catalase-containing solutions, to prevent loss of catalase due to " Complex II " formation (Chance, 1950 ; Adams and Burgess, 1959*b*).

*Tumour.*—Sarcoma 37 was obtained originally from the Imperial Cancer Research Fund Laboratories and maintained by serial passage in this laboratory. Tumour (and normal) tissues were coarsely homogenised in normal saline solution and injected subcutaneously. The doses cited are weights of tissue (as homogenate) contained in the inoculum.

### RESULTS

It was decided at first to study the effects of daily injections of a chemical agent on liver catalase activity and intracellular distribution of catalase. Since (Adams, 1960) animals injected with thiourea had been found to have low catalase activities, a comparison was made of the effects of single and multiple doses of this substance on the catalase activity and intracellular distribution in male mice (Fig. 1). The results show that single doses of 6 $\mu$moles or 10 $\mu$moles of thiourea produced a depression of catalase activity in both granule and extra-particulate cytoplasmic (EPC) fractions without change in the granule/EPC catalase distribution ratio. By the 4th or 5th day the catalase activity had returned to normal. It is evident from Fig. 1 that continuous daily injections of 6 $\mu$moles of thiourea gave results resembling those seen after the injection of the single dose of 10 $\mu$moles. Fig. 2 shows the effect of the continuous daily injection of varying doses of mixed normal liver-spleen-kidney homogenate prepared from 101 strain mice on the catalase activity and distribution ratio of CBA female mice. A daily dose of 25 mg. produced a fall in catalase activity followed by a return to normal by the 4th day. Fifty mg./day produced a greater depression, and the activity began to rise again after three days. Some evidence of a rise in catalase activity from the lowest point was also obtained with a dose of 100 mg./day but since the mice did not tolerate this dose well, the experiment was not continued beyond the 5th day. At no time was there any significant change in the granule/EPC catalase distribution ratio.

A similar experiment was done using a homogenate of Sarcoma 37 tissue. Daily injections of 25 mg. produced little effect on the granule catalase activity, but significantly reduced the EPC level. Fifty mg./day produced depressions of activity in both fractions followed by restoration of the granule catalase activity

FIG. 1.—A comparison of the effects on liver catalase activity and granule/EPC distribution ratio of single and continuous daily injections of thiourea. Doses given at times indicated by ↑. Graph (*a*) 6 μmoles (*b*) 10 μmoles (*c*) 6 μmoles/day. Total catalase (△– – –△), granule catalase (○– – –○) and EPC catalase (●– – –●) activities are given in this and subsequent figures as arithmetic means ± Std. error of mans. Eight animals/group.

to normal by the 5th day, but the EPC catalase remained depressed. The results of the 100 mg./day dose showed a rise from the minimum level by the fifth day. As with the normal tissue, this dose was not well tolerated by the mice. Unlike the normal tissue experiments, the granule/EPC catalase distribution ratio was increased to about 6·0–6·5, and this occurred independently of dose.

In a further experiment male mice were injected daily with 50 mg. doses of homogenised S37, and another group with the same dose of liver-spleen-kidney homogenate. As with the female mice the normal tissue produced a depression in catalase activity without change in the granule/EPC distribution ratio. The rise of catalase activity from the lowest point was more pronounced than in the females receiving the same dosage. An increase in catalase granule/EPC distribution ratio was found in the males treated with S37, and there was a sharp rise in catalase activity from the lowest point. It appeared in this experiment that the distribution ratio also was returning towards normal on the 3rd and 4th days.

### DISCUSSION

The results have shown clearly that while multiple injections of Sarcoma 37 produced a depression in liver catalase level and an increase in catalase granule/EPC distribution ratio the tumour injections failed in a number of respects to

FIG. 2.—The effect of the continuous daily injection of normal tissue (mixed LSK) homogenate on the catalase activity and granule/EPC distribution ratio in CBA female mice. (*a*) 25 mg./day (*b*) 50 mg./day (*c*) 100 mg./day.

simulate the picture in the tumour-bearing mice. Even when the dose was raised to the maximum tolerated by the animals, Sarcoma 37 homogenate failed to increase the catalase granule/EPC distribution ratio beyond 6·0–6·5 in females and 4·9 in males compared with about 8·0 and 6·5 in tumour-bearing females and males. Also the total catalase activity was still 60 per cent of normal as against about 40 per cent or less in tumour-bearing animals (Adams, 1959). The increase in distribution ratio was independent of dose over the range studied.

The experiments with thiourea showed that the catalase activity of treated animals was able to return to normal while the injections were still being given.

This suggests that the rate of catalase synthesis can be increased considerably to meet extra demands on the enzyme. There must however be limits to the ability of the system to restore catalase activity to normal, during the continuous injection of agents which reduce the activity. Thus, although in the tissue homogenate injection experiments there was evidence that catalase activity rose from the lowest point during the injection of doses up to 50 mg./day, it was not clear whether

FIG. 3.—The effect of continuous daily injections of S37 tissue homogenate on the catalase activity and granule/EPC distribution ratio in CBA female mice. (*a*) 25 mg./day (*b*) 50 mg./day (*c*) 100 mg./day.

this occurred during the injection of 100 mg./day. There was, it is true some evidence of a rise from the lowest point, but it was not possible to continue the experiments beyond about 5 days. In any event, however, if the catalase depression in tumour-bearing animals is due to release of a specific tumour agent plus tumour breakdown products it follows that the liberation of at least 100 mg./day of tumour substance would be required to prevent a return of catalase activity to normal. But it seems that not even this dose will alter catalase activity and distribution ratio to the values reached in tumour-bearing animals. If catalase levels following the continuous injection of tumour tissue are compared with those found in tumour-bearing animals (Adams, 1959), 100 mg./day of S37 is equivalent to the presence in the host of a tumour weighing one gram or less. Apart from the fact that 100 mg. of tissue/day was the maximum dose tolerated by the animals, it seems unreasonable to suppose that tumours are capable of releasing this amount of breakdown products per day, especially in the early stages of growth. Further the loss in total catalase activity following the injection of S37 homogenate is if anything less than that following the injection of normal LSK homogenate, leaving little or no room for the contribution of a specific tumour agent. It may be

FIG. 4.—The effects of the continuous daily injection of (*a*) 50 mg. S37 homogenate, (*b*) 50 mg. normal LSK as homogenate, on the catalase activity and granule/EPC distribution ratio of CBA male mice.

argued that the catalase depressing effect of the tumour is mainly due to the release of a specific agent, but as already stated, Kampschmidt, M. E. Adams and Mc'Coy (1959) found that catalase activity rose to normal while tumour fractions were being injected, so that either this agent must be absent from, or present in insufficient amount in such fractions, or some other effect of the growing tumour must be sought for.

One point of interest arising from this paper is that the EPC catalase activity of the CBA males was not decreased to a greater extent than that of females by the injection of tumour homogenate. This contrasts with earlier results reported using an albino strain (Adams, 1950, 1951). Recently (Adams, 1960, and unpublished experiments) evidence has been obtained that different androgenic hormones are present in the CBA and albino strain of mice. CBA female mice injected with testosterone had EPC catalase levels elevated about halfway to the male level. The EPC catalase level of females of the albino strain under the same conditions was elevated to the male level. When 17 methyltestosterone was injected into females of both strains, the EPC catalase activity of the CBAs rose to the male level, and of the albinos to considerably above the male level. In view of the evidence (Adams, 1951) that a tumour agent interferes with the action of hormones on catalase, one explanation of the greater resistance of the catalase in CBA mice may be that they secrete a different androgenic hormone.

It was suggested (Adams, 1959) that the effects on catalase produced by tumour homogenate injections could be divided into two parts : a depression in total catalase activity, which appeared quite non-specific, and a change in catalase distribution. The present work lends additional support to this view, and it may well be asked what relevance the ability of tumour homogenates or fractions to *depress* catalase activity has to the lowered catalase activity in the tumour-bearing animal. It is therefore proposed to continue this enquiry on the supposition that an agent present in tumours induces changes in the host which, in conjunction with certain nutritive demands of the tumour, lead to the observed changes in host catalase activity and distribution.

### SUMMARY

(1) The changes in mouse liver catalase activity and distribution following multiple injections of Sarcoma 37 homogenate resemble, but do not quantitatively match, those seen in S37-bearing mice.

(2) It is concluded that a tumour agent alone can hardly be responsible for the observed changes in tumour-bearing animals.

My thanks are due to Dr. M. H. Salaman for his interest and to Miss Iris Harris for skilled technical assistance. The expenses of this research were partly defrayed out of a block grant from the British Empire Cancer Campaign.

### REFERENCES

ADAMS, D. H.—(1950) *Brit. J. Cancer*, **4**, 183.—(1951) *Ibid.*, **5**, 115.—(1952) *Biochem. J.*, **50**, 486.—(1959) *Brit. J. Cancer*, **13**, 704.—(1960) *Biochem. J.*, **74**, 141.

*Idem* AND BURGESS, E. A.—(1957) *Brit. J. Cancer*, **11**, 310.—(1959*a*) *Biochem. J.*, **71**, 340.—(1959*b*) *Enzymologia*, **20**, 341.

*Idem* AND ROE, F. J. C.—(1953) *Brit. J. Cancer*, **7**, 509.

CHANCE, B.—(1950) *Biochem. J.*, **46**, 387.

KAMPSCHMIDT, R. F., ADAMS, M. E. AND McCOY, T. A.—(1959) *Cancer Res.*, **19**, 236.

THOMSON, J. F. AND KLIPFEL, F. J.—(1958) *Ibid.*, **18**, 229.